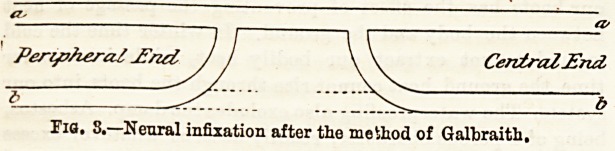# Surgery of the Nerves

**Published:** 1896-01-04

**Authors:** 


### SURGERY OF THE NERVES.

**The Repair of Divided Nerves.**—The repair of nerves divided in their continuity, after varying intervals of time, is one of the procedures which antiseptic methods has brought within the range of practical surgery. A considerable amount of experimental and clinical work has been done on this subject since Flourens first, and Létiévant later, showed the possibility of restoring function in divided nerves. Huber and Howell[1] and others have demonstrated that when a nerve is cut the peripheral end degenerates, and that the degeneration involves both axis cylinder and medullary sheath. They are further of opinion that the function is restored by the down-growing of the axis cylinder process from the central end into the peripheral part of the nerve. Regeneration of the peripheral end they hold can take place in no other way. Hence the importance of obtaining as close apposition of the divided ends as possible in operating.

**Nerve Suture.**—There are two plans on which the repair of divided nerves may be carried out—nerve suture, and nerve infixation. To the former method—nerve suture—Mr. John Duncan, of Edinburgh[2], draws attention in a clinical lecture. After speculating on the physiological problems involved in the operation, he cites a case in which he operated for division of the median nerve four days after the accident. The friends of the patient had refused to have anything done at the time, the wrist was cut with a broken bottle, and the wound was simply dressed antiseptically. The function of the median nerve was entirely abrogated, and on the fourth day the ends were found to be half an inch apart. One stitch was passed through the nerve substance, and two others through the sheath only. After stitching tendons and skin, the hand was put up in the position of flexion. On the third day after operation it was found that the patient could localise touch sensations with perfect correctness, all over the hand, although a little slowly in the median area. In little more than a week there was absolutely no difference in sensitiveness between the injured and the sound hand. After the tendons had healed it was found that the muscles supplied by the median acted quite normally and had undergone no atrophy. Although holding that a much freer anastomosis takes place between sensory branches of nerves than is generally supposed, Mr. Duncan does not think that his case is an example of "supplementary sensation" because of the rapid and perfect restoration of function, and the accompanying motor recovery.

In another case the operation was not sought till five months after injury. A young man divided his posterior tibial nerve, causing complete paralysis and atrophy of the muscles and loss of sensation. On making an incision in the line of the nerve the upper end, B (Fig. 1), was found bulbous and the lower end, C, atrophied, and they were separated by rather more than an inch. It was impossible to approximate the ends without an

FIG. 1.—The upper figures indicate the position before suture, the lower figure after suture. The dotted lines indicate the incision.

amount of straining or dissection, which would have endangered the nerve's vitality. After paring the lower end the bulbous portion was incised from above downwards, and the loosened end turned down and its upper raw end, A, sewed to the peripheral portion of the nerve as illustrated in the diagram.

Three weeks after there was very slight improvement of sensation, and not till six months afterwards did distinct improvement make itself manifest. A year after operation the foot was in every respect as good as the other. This operation is based on the fact that a nerve fibre can carry either sensory or motor impressions in both directions. The writer concludes that such operations are eminently justifiable even after long periods, although the probability of complete success diminishes rapidly after the lapse of a year or two. Aseptic healing is most essential.

Another case of nerve suture is recorded by Sinkler.[3] The patient was stabbed with a penknife, and the musculo-spiral nerve divided three inches above the elbow. The following day there was noticed loss of power in the extensors of the wrist and fingers, and a sensation of pins and needles in the thumb and forefingers, with pain in the arm on movement. When operated on three months later there was some return of sensation, but no improvement in motor power, and there was considerable muscular atrophy, with loss of electrical reaction. The nerve when exposed was found to be bulbous (Fig. 2) at the point where it had been wounded. The bulb was excised and the raw ends, *a* and *b*, sutured with fine silk. Eleven days after the operation, when all was healed, on examination no muscular improvement was present. It was not for eleven months that complete use of the arm had been regained. Clinically the case is of value as showing the great advantage of the operation, even after a long interval, and also the length of time which often elapses before recovery is complete. It was further observed that voluntary power returned long before the muscles reacted normally to electricity. Dr. Sinkler emphasises the value of electricity and massage in the interval between the operation and the onset of evidence of improvement. Pathologically it is interesting to note that the nerve bulb excised at the operation was found to show the development of young nerve tissue amongst the fibrous tissue and neuroglia, and it seems possible that in time this would have been completely restored the function of the nerve.

FIG. 2.

**Neural Infixation.**—Apropos of a case in which he performed the operation of "infixation," Dr. Harvey Reed[4] contributes a paper on this subject. In his own case he slightly modified the method suggested by Galbraith, which consists in implanting both the central and the peripheral ends of a divided nerve into the trunk of a parallel healthy one, so that the nerve impulses shall be carried along the section of the healthy nerve intervening between the points of infixation. (Fig. 3.) By this operation the degeneration of the peripheral end which followed in Létiévant's case, where only that end was implanted, and also in an experiment of Huber on cross-suturing of

FIG. 3.—Neural infixation after the method of Galbraith.

nerves, is obviated. This is due, Reed thinks, to the central and peripheral ends being brought into, at least, indirect connection with one another. In his own case, in the dissection of glands from the axilla, the circumflex nerve and a large muscular branch were both cut. The central ends of the two were connected, and then the peripheral end of the muscular branch was infixed to the central end of the circumflex. The posterior thoracic nerve, which was also cut, was simply sutured. For a considerable time clinical evidence of the division of the nerves was manifest, but gradually disappeared, and nine months later the patient was quite well.

**Operations for Trigeminal Neuralgia.**—In a paper read before the Congress of the German Surgical Society, Krause[5] advocates the operation of excision of the Gasserian ganglion in very severe and intractable cases of this condition. He points out that although no definite histological change has been demonstrated in the branches of the fifth nerve, such lesions do occur in the Gasserian ganglion, and he uses this fact as an argument in favour of extirpating the ganglion in preference to resection of the nerve trunks. No relapse of the neuralgia after ablation of the ganglion has yet been recorded, says Krause. He has never had serious eye symptoms follow the operation, although the cornea and conjunctiva are both rendered completely anæsthetic. Krause performs the operation by raising a flap of soft tissues and bone from the temporal region just above the zygoma. The dura mater and brain are then raised by a broad elevator and the middle meningeal artery tied and divided. The infra-maxillary and supra-maxillary divisions of the nerve are then divided close to their foramina of exit, and the ganglion is seized and twisted out. The ophthalmic division is destroyed at the same time. The flap is replaced and sutured in position. The only serious risk in the operation is hæmorrhage from the dura mater, middle meningeal artery, or cavernous sinus. This is met by pressure, but is sometimes so severe that the second stage of the operation, viz., the extirpation of the ganglion, has to be deferred for a few days. The same difficulty has been met with by Gerster,[6] who reports a case fatal from brain softening as a consequence of the operation. Krause quotes fifty-one cases by different surgeons with five deaths. An additional case in which no recurrence of pain has taken place six weeks after operation is reported by R. W. Stewart.[7] In this case the ganglion was destroyed by a dental excavator.

**Traumatic Neuritis.**—In cases of severe pain in parts after injury, where there is reason to believe that the cause is an ascending neuritis, Delorme[8] has found in eight cases that very firmly compressing the part for a short period (a few seconds) has effected a cure. The painful part is grasped by the surgeon and compressed as firmly as can be. Usually one compression is enough; but, if necessary, it may be repeated after five or six days. Presumably it acts by breaking down adhesions.

1 Jour. Physiol. vols. xiii., xiv. 2 Clin. Journ., Oct. 9th, 1895. 3 Therap. Gazette, July 15th, 1895. 4 Annals of Surgery, Sept. 1895. 5 Centralblatt f. Chirurgie, 27, 1895. 6 N.Y. Medical Record, June, 1895. 7 Medical News, Ap. 1895. 8 Gaz. des Hôpitaux, 1891, No. 1.

## Figures and Tables

**Fig. 1. f1:**
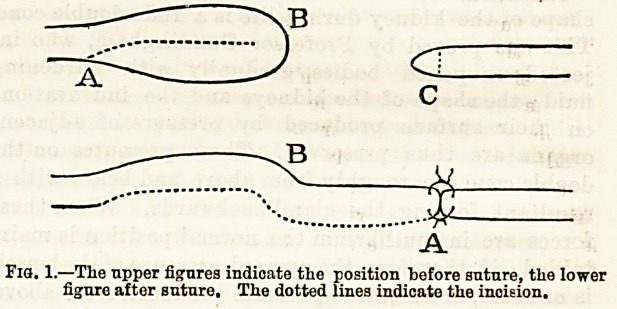


**Fig. 2. f2:**
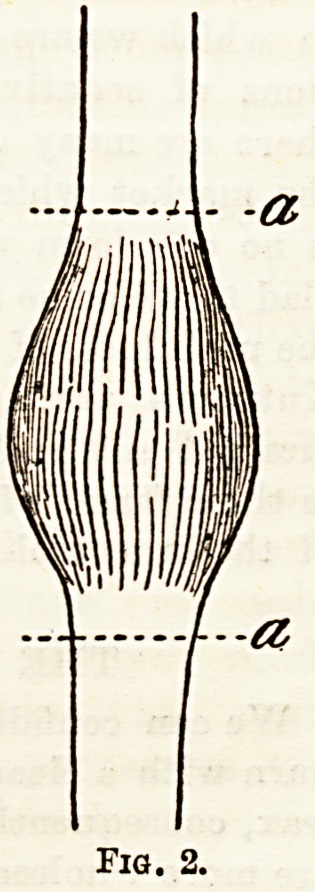


**Fig. 3. f3:**